# ASSESSING BALANCE INTERVENTION OUTCOMES IN OLDER ADULTS WITH HEARING LOSS

**DOI:** 10.1093/geroni/igad104.2274

**Published:** 2023-12-21

**Authors:** Caitlin Price, Holland Hardison, Laura Spradley

**Affiliations:** University of Arkansas for Medical Sciences, Little Rock, Arkansas, United States; University of Arkansas for Medical Sciences, Little Rock, Arkansas, United States; UAMS, Little Rock, Arkansas, United States

## Abstract

Previous studies reveal individuals with hearing loss are more likely to experience falls and fall-related injury. Fall risk exponentially increases with more severe degrees of hearing loss. Because both the risk of falls and the incidence of hearing loss increase with age, investigating the efficacy of balance intervention programs in older adults with hearing loss is vital to enhancing clinical outcomes and reducing heightened risk of falls in these individuals. Various balance intervention and physical exercise programs have been shown to reduce fall rates. However, which individuals achieve the greatest benefit from these programs remains unclear. Do those at greater risk of falls demonstrate more or less benefit from balance intervention? The current study aimed to (1) assess the impact of the A Matter of Balance (AMOB) program on older adults’ fall risk and balance measures and (2) evaluate which measures (e.g., postural stability, balance confidence, fall risk, physical activity, degree of hearing loss) best predict balance intervention outcomes. Pre- and post-intervention balance and fall risk in older adult participants (60-90 years) with hearing loss were assessed via measures of postural sway and subjective questionnaires. Results revealed balance-related benefits following the completion of the AMOB program. Older adults initially identified as having higher fall risk demonstrated the most significant benefits as observed in fall risk reduction. Our results inform clinical practice by clarifying which individuals may benefit most from balance intervention programs and thus improve targeted intervention approaches for older adults at greater risk for falls and fall-related injury.

